# A Contemporary Review of Trachea, Nose, and Ear Cartilage Bioengineering and Additive Manufacturing

**DOI:** 10.3390/biomimetics9060327

**Published:** 2024-05-29

**Authors:** Max Feng, Khwaja Hamzah Ahmed, Nihal Punjabi, Jared C. Inman

**Affiliations:** 1 Department of Otolaryngology–Head and Neck Surgery, Loma Linda University Medical Center, Loma Linda, CA 92354, USA; 2 School of Medicine, Case Western Reserve University, Cleveland, OH 44116, USA

**Keywords:** trachea, nose, septum, ear, reconstruction, cartilage, bioengineering, additive manufacturing

## Abstract

The complex structure, chemical composition, and biomechanical properties of craniofacial cartilaginous structures make them challenging to reconstruct. Autologous grafts have limited tissue availability and can cause significant donor-site morbidity, homologous grafts often require immunosuppression, and alloplastic grafts may have high rates of infection or displacement. Furthermore, all these grafting techniques require a high level of surgical skill to ensure that the reconstruction matches the original structure. Current research indicates that additive manufacturing shows promise in overcoming these limitations. Autologous stem cells have been developed into cartilage when exposed to the appropriate growth factors and culture conditions, such as mechanical stress and oxygen deprivation. Additive manufacturing allows for increased precision when engineering scaffolds for stem cell cultures. Fine control over the porosity and structure of a material ensures adequate cell adhesion and fit between the graft and the defect. Several recent tissue engineering studies have focused on the trachea, nose, and ear, as these structures are often damaged by congenital conditions, trauma, and malignancy. This article reviews the limitations of current reconstructive techniques and the new developments in additive manufacturing for tracheal, nasal, and auricular cartilages.

## 1. Introduction

Craniofacial reconstruction of the head and neck has seen many advancements in the past century. Local-flap and free-tissue transfers have allowed for the reconstruction of large defects with little to no morbidity. Advances in transplantation medicine and techniques have led to the introduction of face and laryngeal transplantation [1,2]. However, defects in the cartilaginous framework of the head and neck, specifically the trachea, nose, and ear continue to be exceptionally difficult to reconstruct. Current surgical techniques and biotechnology are challenged by the complexity of these anatomic regions. The loss of the cartilaginous framework is the largest challenge to the reconstruction of these areas.

Advances in additive manufacturing and bioengineering offer a promising armament to the reconstruction of the head and neck [3]. Recent discoveries in stem cell technology, tissue culturing techniques, and scaffold manufacturing have shown that cartilage manufacturing is a viable treatment option for patients affected by tracheal, nasal, and auricular defects. The advancement of computer-aided modeling and 3D printing can be used in bioengineering to create highly accurate, patient-specific cartilaginous frameworks.

Although there is abundant literature on tracheal bioengineering, there are few reviews on the clinical applications of additive manufacturing for cartilage reconstruction, particularly in the nose and ear. To our knowledge, this is the first review encompassing translational advances in tracheal, nasal, and auricular bioengineering. In this review, we seek to provide an overview of the unique anatomy and reconstruction challenges of these complex sites, describe the current clinical applications of bioengineering in the treatment of craniofacial pathology, and understand the limitations constraining bioengineering to the laboratory setting.

## 2. Tissue Bioengineering

The generation of a bioengineered tissue requires an initial source from which to expand the cells and a scaffold to provide shape and guide cell delivery [4]. A major challenge in cell generation is the harvesting of suitable cartilage cells. Autologous sources are ideal due to the avoidance of needing immunosuppression to prevent autoimmune rejection. The ideal donor site is minimally invasive and carries low morbidity of harvest.

Currently, the focus of tissue engineering involves autologous chondrocytes or mesenchymal stem cells (MSCs). The nasal septum, rib, and knee are sources of hyaline cartilage. The ear contains elastic cartilage and has the lowest donor-site morbidity, but the use of elastic cartilage cells to generate hyaline cartilage is not completely understood. Nasal septal cartilage has similarly low donor site morbidity and possesses similar qualities to tracheal cartilage. Furthermore, the nasal septum contains chondrocytes, epithelial cells, and connective tissue; all of which can be harvested from a small area of septum [4]. Bone-marrow- and adipose-derived stem cells (ADSCs) have promise in their applications in tissue engineering due to their potential for differentiation into chondrocyte cell lines. MSCs can be harvested through needle aspiration of iliac crest bone marrow or adipose tissue, possessing relatively low morbidity [5]. Chondrogenic MSCs have been shown to have similar levels of type II collagen, glycosaminoglycans, and elastin to chondrocytes after in vivo transplantation in an animal model [6]. However, a limitation of using MSCs is the need for the addition of growth factors to promote differentiation, such as TGF-B1 [5,7]. The combination of MSCs and chondrocytes in co-culture has been shown to improve cartilage quality compared to the use of chondrocytes alone [8].

The scaffold provides a framework that facilitates cellular adhesion, differentiation, proliferation, and migration. Without a scaffold for cellular attachment, cells injected directly into the recipient site will become dispersed, leading to inadequate concentrations of the cells necessary for tissue formation [9]. Material selection is important, since the scaffold material must not elicit an inflammatory or autoimmune response, or have toxic breakdown products, all of which could disrupt tissue generation. The material must also have mechanical strength to prevent collapse after being implanted, possess porosity to allow for even distribution of cells and nutrients, and have a degradation rate that matches cartilage growth [10]. Natural scaffolds are generated from donated homologous tissue, which is decellularized to remove all cellular components while preserving the extracellular matrix and angiogenic factors. This is accomplished through a combination of chemical, physical, or enzymatic methods. Removing all immunogenic cellular components can minimize immunogenicity of the donated tissue. However, decellularization can weaken the structure of the scaffold, leading to collapse after implantation [7,9,11].

Several synthetic materials have been studied for scaffold fabrication. Biodegradable materials include polymers (polyglycolic acid, polylactic acid, polycaprolactone), hydrogels (Pluronic F-127, collagen gel), and decellularized allogenic material [10,12,13]. Non-biodegradable materials have also been investigated, including polypropylene and polytetrafluoroethylene [14]. The material is fabricated into a tubular porous structure to provide adequate surface area for cells to attach and proliferate, support nutrient delivery, allow for the clearance of metabolic waste products, and promote angiogenesis. With insufficient pore size and distribution, cells cannot adequately grow to the center of the scaffold and lack the nutrition to sustain life. Pore size has also been shown to impact cellular differentiation; larger pore sizes led to osteogenesis and smaller pore sizes led to osteochondral formation [15].

Scaffold manufacturing options include molding, electrospinning, and 3D printing. Scaffold molding involves designing a mold based on the measurements of the defect to be reconstructed, then casting the chosen scaffold material into the mold. Electrospinning uses the electric field generated by two different electrodes to deposit polymers into ultra-fine fibers, creating a porous product with a large surface area. This increased porosity and surface area is an ideal environment for cells to grow [16]. The 3D printing method uses a computer-rendered model of the cartilage defect derived from a computer tomography scan. The defect is then segmented with computer software and modified after segmentation to accurately fit the size and shape of the defect. The scaffold material is then printed layer by layer, creating highly accurate scaffolds with deviations of less than 1 mm [17,18]. Prior to 3D bioprinting, a customized model is designed for the patient’s needs. This model is then loaded into the 3D bioprinter, which uses bio-ink to create a scaffold layer by layer. Bio-ink is created from seed cells and growth factor additives which maximize biocompatibility and mechanical properties to best replicate cartilage [19].

Tissue engineering requires complex interactions between biomaterials and cells to produce organic structures capable of supporting physiological function when implanted. Bioreactors can provide culture environments that more closely resemble in vivo conditions. By mirroring the physiological and mechanical stimuli of in vivo conditions, bioreactors can achieve larger and thicker cartilage constructs [20]. Dynamic culture systems allow for cell distribution homogeneously across the scaffold, facilitating nutrient supply, oxygenation, and waste removal. These dynamic bioreactors rotate to disperse cells uniformly and generate hydrodynamic forces. They mimic the mechanical forces of the airway, such as shear stress, stretch, and compression, in order to guide proper cell differentiation, extracellular matrix formation, and tissue formation [21]. Two separate bioreactor components allow for the seeding and culturing of chondrocytes or chondrogenic MSCs to the outer surface and epithelial cells to the inner surface [11].

Seed cells must be induced to differentiate into chondrocytes and produce an extracellular matrix (ECM) to create optimal neocartilage. Researchers have found that growth factors, mechanical stress, and oxygen deprivation are the most potent stimuli for MSC differentiation [19]. There are a variety of different growth factors that promote chondrogenic differentiation of MSCs, with the transforming growth factor beta (TGF-B) protein superfamily contributing the most efficacious stimulants. This family of polypeptides includes TGF-B1, B2, B3, and bone morphogenic proteins (BMPs) which are all highly potent stimulators of chondrocyte differentiation [22]. TGF-B1 upregulates the expression of aggrecan, COLII, and SOX9 genes, which are essential for the synthesis and proper functioning of cartilage [23]. Gene transfection using viral factors is an alternate technique for inducing differentiation. Multiple animal studies have shown that the transfection of MSCs with SOX-5, 6, and 9 genes can stimulate differentiation without the need for TGF-B supplementation [24].

Beyond biochemical stimulation, mechanical stimuli have shown strong potential in MSC differentiation. Human cartilage faces mechanical stress on a daily basis; the application of these stresses on MSCs helps replicate physiologic conditions. Mechanical stimulation can be classified as hydrostatic pressure, shear, or compressive force. Each of these forces has been shown to promote chondrogenic differentiation when applied in intermittent cycles [25]. Low-oxygen conditions have been shown to be a particularly potent stimulus as well. The partial pressure of oxygen measured in septal cartilage is substantially lower than the atmospheric oxygen levels in traditional tissue culture incubators. Multiple studies have shown that hypoxic conditions promote chondrocyte differentiation and produce neocartilage with similar biochemical properties to natural cartilage [26,27].

## 3. Trachea

The turn of the 20th century saw a leap in medical innovation and critical care medicine. The development of endotracheal intubation and mechanical ventilation led to the prolongation of life, but also shifted the etiology of tracheal stenosis away from infectious causes, such as diphtheria and syphilis, to iatrogenic causes, namely prolonged intubation and tracheostomy [28]. Injury to the trachea can significantly decrease an individual’s quality of life by impacting breathing, speaking, and swallowing. 

**Figure 1:**
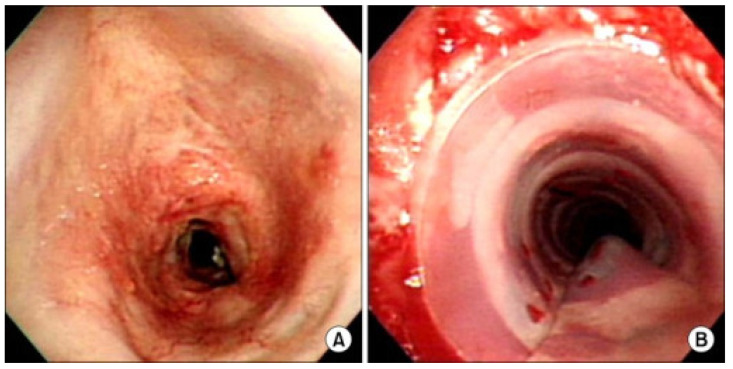
(**A**): Endoscopic bronchoscopy view of a patient with post-intubation tracheal stenosis. (**B**): Endoscopic view after rigid bronchoscopic dilation and silicone stenting. Reprinted under Creative Commons License from ref. [31]. 2022, Creative Commons.

The current standard of care for definitive surgical management of tracheal stenosis is tracheal resection with primary end-to-end anastomosis. However, even with tracheal release techniques, the maximal length of the excised trachea is limited to 6 cm in adults and one-third of the tracheal length in pediatric patients [29]. Tracheal resection also carries significant morbidity and complications can be disastrous. Patients who cannot be addressed with tracheal resection are managed with endoscopic laser excision, balloon dilation, and stenting, which are suboptimal and require repeat surgeries [28,29,30]. Figure 1 demonstrates endoscopic intraluminal views of an airway with tracheal stenosis prior to and immediately after endoscopic rigid dilation with silicone stenting [31]. Various surgical treatments have been attempted to address these issues, including fabricating homologous cartilage and tissue for tracheal reconstruction, as well as tracheal transplantation, which is limited by donor site morbidity, the low supply of donor tissue, and the need for lifelong immunosuppression therapy [11].

**Figure 2:**
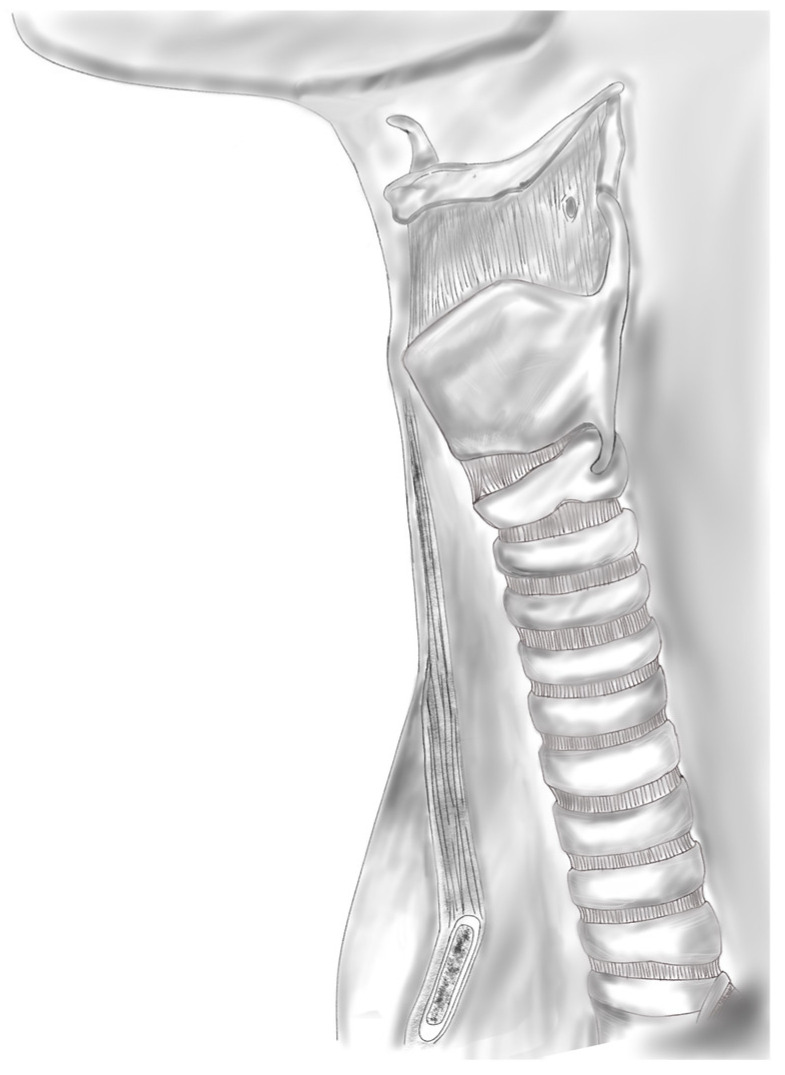
The external framework of the trachea, composed of C-shaped cartilaginous rings separated by an intercartilaginous membrane.

The trachea has two primary functions: to be a ventilatory circuit from the upper airway to the lungs and to clear tracheobronchial secretions. The trachea is composed of an internal lining of ciliated respiratory epithelium and an external framework composed of hyaline cartilage that is not only flexible enough to support the bending and twisting of the neck but is also rigid enough to withstand dynamic collapse during inspiration and high positive pressures during forceful expiration [4,28]. The trachea is unique in that the anterior two-thirds is composed of 18–22 C-shaped cartilaginous rings approximately 4 mm in length, with an intercartilaginous membrane between rings, as shown in Figure 2. The posterior third consists of the trachealis muscle [32]. The trachea is also constantly exposed to the outside environment and must be capable of self-repair, remodeling, regeneration, and resisting infection [33]. Tracheal reconstruction is particularly challenging due to these inherent qualities. Tissue engineering is a promising solution to overcome these limitations. In this section, we will focus on the reconstruction of circumferential tracheal defects.

Decellularized tracheal allografts from deceased donors can be used as scaffolds. The purpose of decellularization is to remove immunogenic cellular and nuclear material from donor tissue while retaining the integrity of the ECM. The ECM—composed of glycosaminoglycan, collagen, proteoglycans, and other glycoproteins—imparts the biomechanical properties to the trachea. The main biomechanical component of the trachea is collagen, which consists of fibers oriented horizontally and longitudinally. This organization imparts lateral rigidity and longitudinal flexibility. The ECM is also essential for intercellular paracrine signaling, intracellular autocrine signaling, and cellular formation by mechanical pressure [34].

Various chemicals have been studied for decellularization. These include acids and bases, which catalyze the hydrolytic degradation of nucleic acids, cytoplasmic components, and biomolecules. Commonly used acids include peracetic acid and hydrochloric acid. Commonly used bases include ammonium hydroxide, sodium hydroxide, and sodium sulfide [35,36]. Organic diluents, which include alcohol, acetone, and 1% tributyl phosphate work through cell membrane lysis [37]. Hypertonic fluid solutions cause cell volume loss and death. Hypotonic solutions cause cell volume overload and membrane lysis. However, these solutions cannot effectively remove DNA from cells. Ionic detergents, which include sodium dodecyl sulfate, sodium dodecyl cholate, Triton X-200, and sodium hypochlorite, are capable of dissolving lipids and removing growth factors in the ECM [37]. Non-ionic detergents disrupt the cell structure by destroying lipid–lipid and lipid–protein bonds without destroying protein structures and glycosaminoglycan, but can potentially reduce the concentration of laminin/fibronectin in the ECM. Enzymatic agents work by breaking peptide bonds between proteins, breaking RNA and DNA bonds, separating basement membrane components from the epithelial layer, or damaging phospholipid components. These include trypsin, exo/endonuclease, dispase, and phospholipase A2 [37].

Conconi et al. reported the feasibility of a detergent–enzymatic method for obtaining the adhesion of chondrocytes and tracheal epithelial cells in a decellularized porcine trachea model in vitro. The trachea was initially rinsed in phosphate-buffered saline containing a dilute antibiotic and antimicrobial solution. The specimen was then processed with distilled water for 72 h at 4 °C, 4% sodium deoxycholate for 4 h, and 2000 kU Dnase-I in 1 M NaCl for 3 h. Hematoxylin–eosin and immunohistochemical staining was performed to verify the presence of retained cells after each cycle. Eighteen cycles were needed to remove MHC class I and II cells in the tracheal model [38].

A drawback of decellularized tracheal scaffolds is their non-porous structure, preventing cells from detaching from or penetrating the ECM. Recently, a laser micropore technique has been used to increase porosity in decellularized tracheas while retaining the tubular structure and causing only minor ECM damage. The adherence rate of cells was significantly improved [39]. Chondrocyte seeding and culture demonstrated mature tubular cartilage with ECM content and mechanical strength similar to native trachea tissue [40].

Three-dimensional bioprinting is a promising solution to scaffold creation. The scaffold material (PGA, PLA, PCL, PLCA) and cell-containing hydrogels (alginate or gelatin) are printed layer by layer [41]. Gao et al. 3D printed a biodegradable PCL scaffold which was cultured with chondrocytes for 2–4 weeks. This was implanted into the subcutaneous tissue of nude mice and demonstrated properties of mature tracheal cartilage. The implanted engineered tracheas demonstrated the feasibility of 3D printing [42]. Similar studies were found to be feasible in rabbit and goat models, with robust compressive strength and epithelial tissue formation observed [43,44]. Bae et al. 3D printed PCL scaffolds seeded with rabbit bone-marrow-derived MSCs and respiratory epithelial cells in chondrogenic media and found a similar structure of the bioprinted model to native trachea [45]. However, a major challenge of 3D bioprinting is fabricating tracheal implants with both macroarchitecture that is mechanically sound and microarchitecture that promotes adequate cell migration and proliferation.

Despite these biotechnological advances seen within animal models, human transplantation of a bioengineered trachea is exceedingly difficult and has not shown long-term viability. The trachea is unique in that it is vascularized by a plexus of small blood vessels, rather than a vascular pedicle, which does not allow for direct microvascular anastomosis. Tracheal transplantation studies have shown that the revascularization of an implanted donor trachea can take weeks to months [46]. The posterior membranous portion of the trachea is thought to be most susceptible to avascular necrosis due to the higher metabolic demands of the muscular tissue compared to the anterior cartilage [47]. In attempts to overcome this hurdle, revascularization in the recipient’s tissues, such as the omentum, the forearm, and the sternocleidomastoid, have been attempted [4,48,49]. This two-stage approach is thought to allow for an initial period of revascularization of the allograft prior to transplantation within the tracheal defect. However, the literature on heterotopic revascularization is mostly limited to cases of allogenic tracheal transplantation, not bioengineered tracheal replacement. Significant controversy surrounds this concept. The idea that cells applied to an avascular or synthetic scaffold can regenerate to form complex tissue is thought to be impossible by critics [50].

The first human transplantation of a bioengineered trachea was reported by Macchiarini et al. in 2008. The recipient was a 30-year-old woman with end-stage bronchomalacia. The authors utilized a single-stage approach and used a modification of the detergent-enzymatic method reported by Conconi. They reported that 25 cycles were required to adequately remove immunogenic material while also maintaining the integrity of the ECM. The decellularized scaffold was seeded with autologous chondrocytes and MSCs, then expanded in a bioreactor prior to the replacement of the patient’s left mainstem bronchus [51]. Long-term follow results at 5 years reported recurrent cicatricial stenosis at the anastomotic site, requiring repeated stenting [52]. Further patients who underwent tracheal replacement by Macchiarini suffered from recurrent stenosis or devastating complications. The original papers were ultimately retracted due to scientific misconduct, falsification data, and concern that the tracheal transplantation caused patient harm [53,54].

Elliot et al. reported the use of a bioengineered trachea consisting of a 7 cm segment of decellularized tracheal allograft seeded with MSCs from the recipient’s bone marrow. The recipient was a 12-year-old boy with long-segment congenital tracheal stenosis and pulmonary sling maintained with metal stents. Due to the recipient’s tenuous aorto-tracheal fistula, the MSCs did not have time for expansion in a bioreactor prior to transplantation. Topical human erythropoietin and TGF-B were applied to support angiogenesis and chondrogenesis, respectively. The authors used a pedicled omental flap rotated into the mediastinum to provide a vascularized supporting layer to the tracheal allograft. However, due to the segmental collapse of the graft, the patient required repeated stenting and balloon dilation. The authors note that there was inadequate cartilage regeneration throughout the graft. Bronchoscopy performed at 15 months after surgery showed complete epithelialization, and the cytology of tracheal brushings demonstrated a viable, ciliated respiratory epithelium [30]. A subsequent attempt at synthetic tracheal replacement in a 15-year-old child by Elliot et al. utilized a decellularized allogenic trachea that was seeded with bone-marrow-derived MSCs and nasal respiratory epithelial cells and then expanded in a bioreactor for 48 h. Unfortunately, a sudden airway obstruction, thought to be caused by acute intrathoracic bleeding, led to the patient’s death [55].

## 4. Nose

The nose is the focal point of the face, and is an important landmark in facial aesthetics in addition to its essential role in olfaction and respiratory physiology. It consists of three layers: a soft tissue envelope, an osseocartilaginous framework, and the nasal lining. The upper third is supported by the bony vault, which consists of the paired nasal bones and ascending process of the maxilla bilaterally [56]. The cartilaginous vault supports the lower two-thirds of the nose. The junction of the upper lateral cartilage (ULC), nasal bones, and septum contribute to the keystone region, an important load-bearing region, the disruption of which can lead to mid-vault collapse. The lower lateral cartilages (LLCs) contribute to the lower third of the nose and consist of the medial, middle, and lateral crura. The septum is integral to the internal anatomy of the nose. Posteriorly, the septum is bony, consisting of the perpendicular plate of the ethmoid superiorly and the vomer inferiorly. The cartilaginous septum extends from the bony septum posteriorly, maxillary crest inferiorly, and joins with the ULC dorsally [57]. 

Damage to the underlying nasal framework can lead to disruptions in nasal function and result in significant aesthetic compromise. The etiology of damage can be from trauma, surgery, congenital malformation, or cancer. The most common abnormality of nasal cartilage is a deviated nasal septum, often resulting in nasal obstruction. Surgery to repair the septum can include septoplasty or functional rhinoplasty, depending on the location of abnormalities along the septum. Aesthetic rhinoplasty is used to modify the nasal cartilages in order to achieve a more harmonious nasal shape [58]. Roughly 36% of nonmelanoma facial cancers occur on the nasal ala, requiring reconstruction to replace missing structural components. For the aforementioned surgeries, cartilage grafting can be used to modify, repair, or recreate the nasal framework, depending on the level of injury [59]. 

**Figure 3:**
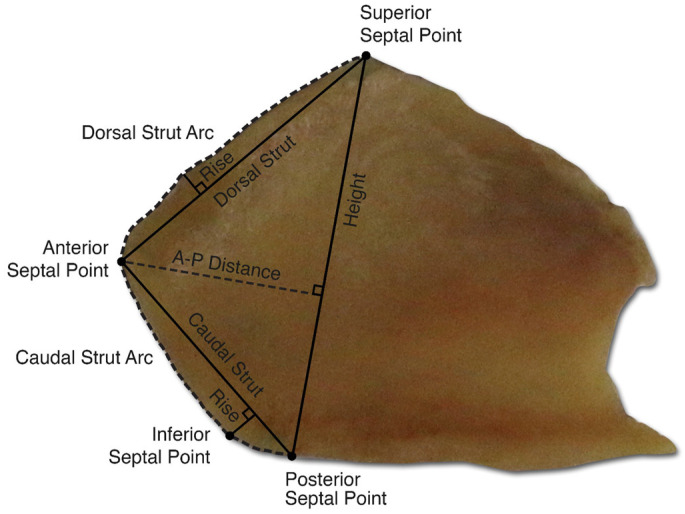
Anatomic photo of nasal septal quadrangular cartilage demonstrating key anatomic points and relationship of dorsal and caudal L-strut to key points.

In primary nasal surgery, the middle portion of the quadrangular cartilage is harvested to remove areas of deviation into the nasal airway and for grafting. A caudal and dorsal L-strut of 1–1.5 cm is left to provide structural support to the nasal tip and dorsum and maintain adequate projection of the nose, as depicted in Figure 3. However, the over-resection of the nasal septum and failure of the L-strut can cause significant structural deformity, such as saddle-nose deformity. In patients with previously operated noses, there is often a paucity of septal cartilage. Revision surgeries are particularly challenging due to this lack of available septal cartilage. In these cases, additional sources of cartilage, typically autologous or homologous, are used for nasal reconstruction.

**Figure 4:**
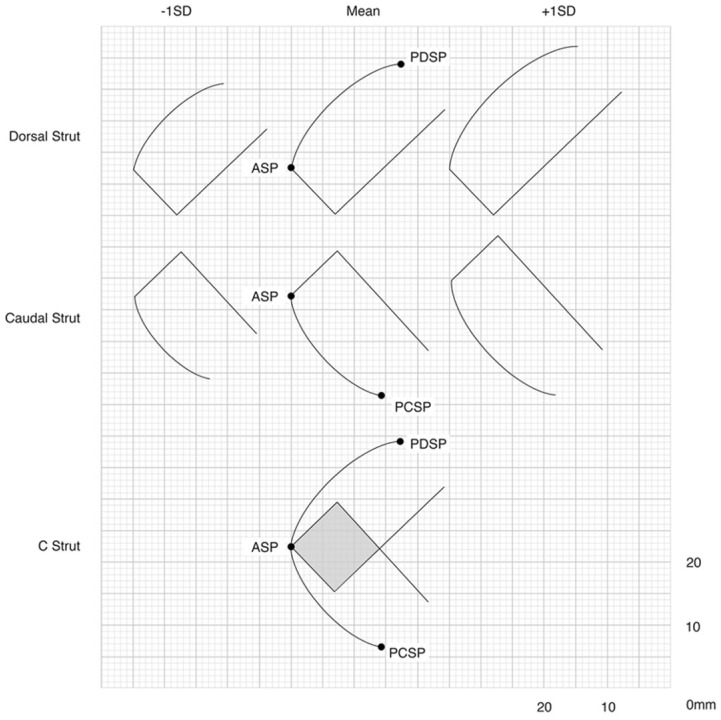
Cross-sectional diagram of the mean, −1 SD, and +1 SD amounts of cartilage required for the reconstruction of the dorsal and caudal septal L-strut.

Autologous grafts are the current gold standard for cartilage grafting [60]. The nasal septum, auricular concha, and rib are the most common donor sites for cartilage harvesting. Autologous grafts are biocompatible and thus have low rates of infection or extrusion. However, these grafts are limited by tissue availability and potential morbidity at the donor site. A significant amount of cartilage is required for reconstruction of the L-strut, as shown in Figure 4. Rib cartilage is more abundant than septal cartilage. However, costal cartilage harvesting does carry a risk of complications, such as increased pain and pneumothorax. For patients with low autologous cartilage availability, homologous cartilage can be used as an alternative. Irradiated costal cartilage from cadaveric donors can be used and provides comparable support to autologous costal cartilage. Additionally, it is purchased prior to surgery and does not require harvesting, thus reducing operating time. However, it does have a higher rate of resorption and is costly. 

Alloplastic implants are an alternative to traditional biological grafts. These implants are made of synthetic material and thus have no associated donor-site morbidity. In addition, they allow for precise carving and do not have a risk of resorption. However, alloplastic implant use is still controversial due to increased rates of infection and extrusion. Silicone implants were one of the first widely used alloplastic implants. Silicone is particularly easy to carve, inexpensive, and has high biocompatibility. Silicone is a non-porous structure, limiting tissue growth and integration and instead relying on the formation of a thick fibrous capsule to fix it in place. Silicone should be placed under the periosteum of the nasal bone, with superficial placement creating a high risk for displacement [61]. However, the capsules created by silicone implant use can contribute to high rates of infection, as they create a dead space for bacteria and limit the inflow of antibiotics [60]. Of the alloplastic implants, silicone has the highest rate of complications and highest rate of removal due to complications, at 12% and 13%, respectively [62]. In contrast, expanded polytetrafluoroethylene has relatively low rates of complication, with multiple studies reporting an overall rate of complication ranging from 3 to 5% [63,64]. Gore-Tex is the most commonly used expanded polytetrafluoroethylene implant. Its microporous structure allows for the ingrowth of host tissues, allowing for implant fixation and limiting displacement. Medpor, a promising high-density polyethylene implant, shows low infection rates between 0 and 6.5% with strong aesthetic results and high satisfaction among patients undergoing rhinoplasty.

Tissue engineering is a promising technology that addresses the scarcity and morbidity of autologous grafts while minimizing the risks of infection associated with implants. Generally, based on requirements for primary rhinoplasty, a tissue-engineered neocartilage would mimic septal cartilage in regard to thickness, microstructure, and mechanical properties. However, revision rhinoplasty and nasal reconstructions tend to use more varied cartilage grafts, thus neocartilages best serve a surgeon by being fabricated in an assortment of thicknesses [58]. Nasal cartilage cellular structure can be organized into peripheral, intermediate, and central zones and tends to vary between the septum, ULC, and LLC. For example, the LLC has denser cellularity in its central zone than the septum and ULC, properties which should be replicated based on which type of cartilage is meant to be replaced by engineered tissue [65]. 

Conventional 3D bioprinting can be used to create neocartilage with homogenous thickness [11]. However, as previously described, nasal cartilage has varied cellularity in its peripheral, central, and intermediate zones. When approaching a deeper zone, there is less cellular density, but cells are larger in size. Newer studies have attempted to create layered neocartilage, but so far none have been conducted for nasal cartilage, with only articular neocartilage having successfully been created with an anisotropic design [66]. Nasal cartilage structure is different from articular cartilage in that it has a perichondrium and differing zones of cellularity. However, both are still hyaline cartilage, and as such, techniques to create anisotropic articular cartilage can still be used for creating layered nasal neocartilage. 

Recent in vivo animal studies have examined the ideal culture composition for bioengineering nasal cartilage. The histologic assessment of glycosaminoglycan (GAG) deposition and type II collagen allows researchers to assess the yield of chondrogenesis after in vivo subcutaneous scaffold implantation into mice models. Apelgren et al. and Moller et al. examined whether scaffolds cultured with septal chondrocytes, MSCs, or both were superior. Surprisingly, both studies found that MSC cultures were not associated with chondrogenesis. Moller found that chondrogenesis was highest in the co-culture group of septal chondrocytes and MSCs and lowest in the monoculture with MSCs. It was theorized that although MSCs may not be able to differentiate into chondrocytes with appropriate factors, they themselves may release certain factors which potentiate cartilage formation by the co-cultured septal chondrocytes. Apelgren’s results contrasted slightly in that there was increased GAG in septal monocultures, despite co-cultures of septal chondrocytes and MSCs having increased chondrocytes. Albeit with some differences, these results still support the theory that MSCs were unable to differentiate into chondrocytes without the necessary factors and septal chondrocytes are instrumental in neocartilage formation [67,68].

A more recent study by Lan et al. highlighted the practical application of tissue engineering for nasal reconstruction. The researchers engineered scaffolds made of collagen 1 hydrogel and septal chondrocytes with the goal of assessing their strength at 3, 6, and 9 weeks of in vitro culture. Of note, these cultures were supplemented with TGF-B, unlike the previous two studies. The constructs were found to be able to withstand the placement of a single 5-0 prolene suture at 9 weeks of in vitro culture. Subsequent in vivo implantation found that the bioengineered tissue continued to strengthen after implantation [69]. This has implications for the surgical timing of bioengineered tissue, as the neocartilage would need to be strong enough to withstand suturing during reconstruction with the understanding that the tissue would continue to mature post-operatively.

In contrast to the abundance of animal studies examining tissue engineering for nasal reconstruction, human studies are still few. In 2014, Fulco et al. conducted a small observational human trial using bioengineered tissue cartilage for nasal reconstruction in melanoma. Five patients who underwent melanoma resection involving the LLC or ULC had bioengineered tissue implanted in the reconstruction process. Autologous chondrocytes from the nasal septum were harvested, expanded, and cultured until the neocartilage was ready for implantation. All five patients underwent the procedure without any complications and were content with functional and aesthetic outcomes 1 year after surgery [59].

## 5. Ear

Ear reconstruction is particularly challenging, in part due to the complex anatomy of the auricle. The outer ear is composed of a single piece of cartilage surrounded by a thin skin envelope. The elastic cartilage is invested by the perichondrium and is of uniform thickness, with numerous folds and valleys that combine to result in the familiar shape of the ear. The anterior topography of the auricle is concave overall, with the posterior aspect being convex [70]. The surface anatomy of the ear includes prominent raised landmarks such as the helix, antihelix, tragus, antitragus, and the superior and inferior crus, as well as the corresponding depressions: the triangular fossa, scaphoid fossa, concha cavum, and concha cymba. Below the antitragus is the lobule, which lacks cartilage and is made of connective tissue and fat. The pathology of the auricular anatomy is difficult to treat, owing mainly to its complex cartilaginous structural framework, which must be recreated accurately to achieve optimal cosmetic outcome.

Microtia refers to a spectrum of congenital malformations involving the underdevelopment of the auricle. The severity of microtia is graded on a scale from I to IV, with grade I microtia representing a smaller ear with intact structures, grade II describing helical deficiencies, grade III being auricles with no recognizable structures, and grade IV being anotia, or a complete lack of auricle. The reported incidence of microtia ranges from 0.83 to 17.4 per 10,000 live births. Males are 2.5 times more frequently affected than females. Most affected individuals have unilateral microtia, ranging from 77 to 93%. 

There are three broad clinical approaches to microtia repair: prostheses, auricular reconstruction with synthetic implants, and auricular reconstruction with autologous costal cartilage. The prosthetic approach involves using a mold modeled after the contralateral normal ear to create a silicone prosthesis—this approach is used in scenarios when patients have failed auricular reconstruction [71]. Auricular reconstruction with alloplastic implants has been shown to have some promise. The first implants were made of silicone and initially had strong outcomes; however, in the long term, they were subject to high rates of infection and extrusion. Medpor implants are superior to silastic implants, yet the gold standard for auricular reconstruction remains autologous cartilage.

**Figure 5:**
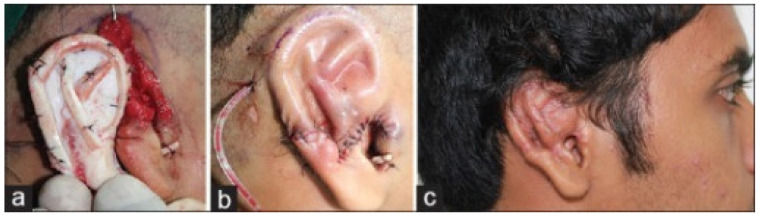
Staged reconstruction of auricular defect using costal cartilage. (**a**): Costal cartilage framework. (**b**): Cartilage framework tunneled subcutaneously and inset. Skin is contoured over the framework. (**c**): Postoperative result after first stage. Reprinted under Creative Commons License from ref. [74]. 2015, Creative Commons.

Autologous ear reconstruction (AER) was initially developed as a six-stage surgery consisting of harvesting costal cartilage from the sixth, seventh, or eighth ribs to sculpt a cartilaginous framework that was implanted beneath the auricular skin [72]. There have been significant advancements in the surgery since its inception and it is now commonly performed as a two-stage rather than a six-stage procedure. However, AER still has significant limitations, with the results of surgery being highly dependent on the artistic and technical skills of the surgeon. The complex structural framework of the ear is difficult to replicate and can be further impacted by the grade of microtia, skin, and cartilage characteristics of the patient, as depicted in Figure 5 [73,74]. 

Bioengineering offers a promising alternative to current methods of auricular framework reconstruction by avoiding donor site morbidity with costal cartilage harvest or the disadvantages of MedPor implant usage. Bioengineering for total or near-total auricular reconstruction requires a safe and readily available cell source, a bioreactor for efficient cell culturing, and scaffolds favorable for cell attachment, proliferation, differentiation, and chondrogenesis.

Perichondrocytes, derived from the perichondrium of the ear, have been shown to be able to differentiate into chondrocytes capable of chondrogenesis due to the presence of cartilage progenitor cells [75,76]. An advantage of using perichondrocytes is the ease of harvest and lack of need for ear cartilage harvest, preserving the framework of the donor ear. In animal models, perichondrocytes cultured in insulin-like growth factor-1 (IGF-1) and transforming growth factor B2 (TGF-B2) showed the production of type II collagen and glycosaminoglycan, which was more pronounced in the perichondrium from younger animal models. Furthermore, the perichondrium has demonstrated superior chondrogenesis compared to MSCs [76]. A drawback of the perichondrium is that its ability for chondrogenesis is influenced by various factors, such as age and culture conditions. In rabbit models, younger rabbits demonstrated a five-fold-increased cartilage yield compared to older rabbits [77].

The ideal scaffold for auricular bioengineering must possess qualities similar to those mentioned earlier, such as being able to induce cell proliferation and migration, being permeable for the flow of nutrients and waste products, and being non-toxic and non-immunogenic. The scaffold should ideally maintain its structure for 2–3 weeks, during which time sufficient ECM generation occurs, so the physical structure of the auricular framework can be supported. Various materials have been studied for scaffold creation. Polylactic acid and polyglycolic acid polymers have been used as synthetic materials for cartilage formation, as mentioned in the previous sections [78]. Several non-synthetic materials have been investigated for auricular scaffold reconstruction. The proteins studied include collagen, gelatin, keratin, and fibronectin, while the polysaccharides studied include alginate, cellulose, hyaluronic acid, chitosan, and glycosaminoglycan. Human fibrin has been shown to form physically stable three-dimensional scaffolds when mixed with cultured chondrocytes. Furthermore, fibrin is highly porous, facilitating nutrient delivery. Fibrin can also be harvested from the patient’s plasma and used autologously, eliminating the risk of immune rejection and disease transmission [79,80]. A photopolymerizing hydrogel system, using in vitro chondrocytes encapsulated in poly(ethylene oxide)-dimethacrylate and poly(ethylene glycol), has also been demonstrated to have increased proteoglycan and collagen contents, along with the presence of a functional ECM [81]. Polyurethane (PU) is a favorable material for auricular reconstruction due to its high strength, flexibility, ease of processing, and low cost. PU can be 3D printed using digitized CT renderings into compression-molded scaffolds for auricular reconstruction. To offset fibrotic capsular contraction of implants, PU scaffolds were coated in hydroxyapetite (HA). The HA-coated PU demonstrated the improved cell number and metabolic activity of fibroblasts [82]. Chitosan, a polysaccharide sourced from the outer skeletons of shellfish, molds, yeasts, and mushrooms, has demonstrated biocompatibility and ability to maintain its structures in vivo, making it a promising scaffold material. Chitosan–scaffold complexes demonstrated relatively low initial type II collagen formation at 4 weeks but displayed progressively stronger protein formation up to 24 weeks out [83,84,85]. Platelet-rich plasma (PRP) has been used as a gelled injectable scaffold, which demonstrated even distribution of chondrocytes and good nutrient exchange. Furthermore, PRP is more biocompatible and less immunogenic in immunocompetent individuals [86].

Another limiting factor in auricular cartilage engineering is the inherent lack of sufficient numbers of autologous chondrogenic cells. Due to the low initial number of cells obtained from a cartilage biopsy, a 300–500-fold expansion of cell numbers is needed for adequate auricular tissue engineering. Initial cell numbers are also limited by the low cellularity of cartilage. Chondrocyte expansion has also been observed to cause dedifferentiation and loss of chondrogenic properties. Different methods to produce autologous chondrogenic cells in large quantities have been studied. The utility of primary passage 0 (P0) chondrocytes have been studied, which when mixed with dedifferentiated chondrocytes (P2), appear to undergo redifferentiation, forming higher levels of type II collagen and proteoglycan than by P2 cells alone [87,88]. However, these studies have been limited to articular cartilage. Other studies have shown that adding basic fibroblast growth factor (bFGF) to the culture medium prevents chondrocyte dedifferentiation during cellular expansion [89,90].

Different methods have been used to generate the unique three-dimensional structure of the human auricle. These include custom molds, external stenting, and computer-aided stereolithography. The first report of a tissue-engineered auricle was from Cao and Vacanti. The authors seeded chondrocytes obtained from the glenohumeral and humeroulnar joints of calves onto a biodegradable polyglycolic acid scaffold and implanted the seeded scaffolds into a subcutaneous pocket on the dorsum of athymic mice. The mice were divided into two experimental groups: those with chondrocyte-seeded scaffolds with an external stent and those without a stent, and a control group without chondrocyte seeding. The stents were applied for 4 weeks. At 12 weeks, the stented experimental specimens demonstrated remarkable likeness to the structure of a human ear, with robust neocartilage formation, while the non-stented group developed rudimentary likeness to a human ear. The control group did not exhibit neocartilage formation [91]. The findings point to the need for external shaping in order to mold neocartilage formation into complex 3D structure of a human auricle. Kamil et al. created scaffolds consisting of PGA and poly-L-lactic acid formed into auricular-shaped silicone molds. After 12 weeks of in vitro growth, the gross structure of the construct resembled a human ear and histological examination showed neocartilage formation [92].

Auricle models have also been created from computer-aided stereolithography, in which a patient’s contralateral normal ear is used as the basis of the model and a liquid UV-sensitive resin is polymerized into the desired structure. The advantage of computer-aided modeling is that an exact mirror image of the patient’s normal ear can be used to fashion the construct [93,94].

Zhou et al. reported one of the first attempts at using a prefabricated, bioengineered auricular cartilage framework for microtia repair. A scaffold, composed of PCL, PGA, and PLA, was compression-molded in a 3D-printed silicone model of the patient’s unaffected ear. The scaffold was seeded with chondrogenic cells and expanded in a chondrogenic medium of Dulbecco’s modified Eagle’s medium, TGF-B1, and IGF-1 for 12 weeks. The bioengineered framework was implanted between the skin and random postauricular fascia flap. At 6 months post-implantation, a cartilage biopsy demonstrated GAG deposition and robust collagen type II expression similar to the native ear. Elastin was also detected in the implanted cartilage, indicating elastic cartilage formation [95]. The authors reported four additional similar cases without evidence of cartilage absorption or extrusion at long-term follow-up.

## 6. Current Limitations and Future Directions

Sadly, research surrounding bioengineered tracheal replacements has been littered with scientific misconduct and a lack of ethical oversight [50,53,54,96,97,98]. A retrospective review of three patients implanted with a total of four synthetic tracheal grafts seeded with bone-marrow-derived MSCs at the Karolinska University Hospital in Sweden revealed that every graft failed to revascularize and epithelialize. Graft-associated complications included fistulas at anastomotic sites, obstructive granulation tissue formation, tracheobronchial–pleural–esophageal–mediastinal fistula formation, and total graft dehiscence. Furthermore, each patient developed major thromboembolic events, which were attributed to the off-label supratherapeutic administration of epoetin in the post-operative period [99]. Critics of tracheal regeneration point to the lack of inherent lack of blood supply to synthetic tracheas, leading to inevitable failure after transplantation. Furthermore, critics challenge the scientific theory that cells applied to a synthetic tube have the intrinsic ability to generate functional tissues and transform from a synthetic graft to a living, functional organ, despite the application of growth factors [50,99,100]. The respiratory tract is also constantly exposed to the outside environment, and synthetic grafts immediately become contaminated once implanted into the airway, which is believed to inhibit cellular ingrowth and contribute to anastomotic failure, granulation tissue formation, and eventually restenosis or dehiscence. This is further supported by the lack of studies reporting the long-term success of synthetic respiratory, gastrointestinal, or genitourinary tract implants in humans. The application of airway stents is also a confounding variable, as the studies reporting long-term outcomes (e.g., more than 3 months) utilized airway stents to preserved the patency of the airway lumen due to granulation tissue formation and restenosis [49,99]. The use of the recipient’s vascularized tissue, such as omentum, to wrap the bioengineered graft may also have simply served to bolster the anastomotic sites and delay the inevitable complications of wound breakdown [100]. A major limiting factor in human studies is the inability of investigators to directly visualize the implant and assess the degree of healing and neocartilage formation. Although endoscopic visualization can assess the epithelial surface of the implant, the degree of cartilage formation cannot be readily discerned. Computed tomographic images in current studies do not clearly depict the amount of tissue formation or show the graft with an airway stent in place [49,99]. As such, further work is needed to develop methods to serially assess the graft in its entirety after implantation.

The concept of a bioengineered tracheal graft for the treatment of long-segment circumferential tracheal defects sounds promising in theory. However, there is still a large gap between laboratory studies and translation to clinical practice. There are currently no solutions that successfully address the challenge of revascularization. A major question must be asked—how can an inherently avascular, synthetic scaffold provide adequate vascular supply to support cellular adhesion, growth, migration, and reorganization? Attempts at overcoming this hurdle, such as cellular expansion in a bioreactor and wrapping the graft in omental tissue, have not been successful. There is much work to be done when investigating novel solutions to achieve adequate vascularization throughout the entire scaffold. The second major question is how can a population of cells applied to a synthetic tube subsequently organize themselves into a complex organ? The trachea is an amazingly complex organ that is far more elegant than a simple tubular structure that air passes through. No human studies have demonstrated that seeded cells can organize into a multilayered structure capable of regeneration, self-repair, remodeling, and resistance to infection. Further research should also focus on the role that growth factors play in influencing cell migration and organization once implanted. Nonetheless, as our understanding of MSCs, tissue culturing, cell seeding, scaffold development, and revascularization strategies grows, bioengineered tracheal transplantation may be a viable treatment option for patients afflicted by tracheal stenosis in the future.

The application for bioengineering in nasal reconstruction is promising; however, there are still limited data on human patients. There are currently no FDA guidelines on bioengineered nasal cartilage, and an optimal combination of biomaterials for scaffold construction and culture makeup has not been finalized [58]. However, the recent literature has shown that bioengineered nasal cartilage is feasible and robust enough to withstand handling during surgical reconstruction. The nose has a complex structural anatomy requiring a variety of different cartilage shapes and thickness depending on the type of defect. Autologous grafts are an ideal source of cartilage but are morbid and limited in quantity. Novel techniques using implants and tissue engineering are being researched with strong potential to allow for the creation of neocartilages, which can be designed for specific patient needs. Beyond nasal cartilage engineering, researchers are attempting to create biologically functional nasal cartilage with an integrated electrochemical sensing system to bring functional olfaction in addition to structural reconstruction [101]. Tissue engineering is a burgeoning field with an exciting future of potential applications for reconstructing the structure and function of the nose.

Cartilage manufacturing for total or near-total auricular reconstruction yields a promising solution to the current multi-stage approach of costal cartilage-based framework reconstruction, which carries significant morbidity and often yields unsatisfactory results. Recent advances in biomaterials and tissue culture research highlight the future possibility of a single-stage approach to auricular reconstruction, in which a prefabricated, highly precise replica of a patient’s contralateral ear is used for framework reconstruction. However, several limiting factors remain. A large expansion in chondrogenic cell numbers is required prior to implantation. The use of growth factors, such as bFGF, has shown promising results in preventing cell dedifferentiation during expansion. Further work is needed in investigating optimal tissue culture methods for generating chondrogenic cells on a large scale prior to widespread use in humans. There is also a paucity of long-term human studies. The long-term resilience of implanted bioengineered cartilage in order to maintain its rigidity and withstand repetitive forces and trauma while also remaining flexible is not known. Initial human studies show that the implanted cartilage continues to remodel and change its collagen-to-elastin content [95]. Lastly, the effects of scaffold breakdown products are not well understood in humans. Open-pored polyurethane scaffolds have been shown to release dextrose, triethanolamine, and poly(ethylene glycol)-*block*-poly(propylene glycol)-*block*-poly(ethylene glycol) and could have potential cytotoxic effects on human chondrocytes and lymphocytes [102]. Further research is needed to investigate the potential breakdown products of biodegradable scaffold material and the potential effects on graft integrity and human safety.

## 7. Conclusions

Tissue engineering and additive manufacturing holds the potential to allow for the reconstruction of complex craniofacial defects that autologous reconstruction methods are limited in. Table 1 summarizes the limitations of the current treatments and the novel additive manufacturing techniques that are being developed for tracheal, nasal, and auricular cartilage. The use of autologous chondrocytes and stem cells avoids the immunogenic challenges faced in allogenic tissue transplantation, as well as the issues with implant extrusion and infection with alloplastic implants. The ear and nasal septum are readily available sources of cells for chondrogenesis. Adipose tissue and bone marrow also serve as sources of MSCs that have minimal morbidity of harvest. However, most of the recent advances in tissue engineering have been limited to in vitro and animal studies. There are very few human studies on bioengineered tissue implantation. Although more work has been carried out in the field of bioengineered trachea implantation, human studies have been marred by lack of graft failure and significant patient morbidity. Challenges with adequate revascularization and tissue growth severely limit their utility and safety for human use. Initial studies on nasal and auricular reconstruction with bioengineered grafts have shown efficacy, but no long-term data on the viability and safety of the implanted cartilage exist. Another limiting factor is the small amount of neocartilage generated with current techniques. Translation to large-scale neocartilage production is required before being widely available for clinical use.

## Figures and Tables

**Table 1:** Comparison of additive manufacturing methods, uses, and limitations in tracheal, nasal, and auricular cartilage.

	Tracheal Cartilage	Nasal Cartilage	Auricular Cartilage
Biomechanical Properties	Flexible yet rigid hyaline cartilage capable of withstanding dynamic collapse	Multiple segments of hyaline cartilage with varying levels of thickness and rigidity	Single piece of elastic cartilage of uniform thickness but highly varied topography
Cell Sources	Autologous chondrocytes and MSCs	Autologous chondrocytes and MSCs	Autologous chondrocytes, perichondrocytes, and MSCs
Scaffold Materials	Decellularized donor trachea, PGA, PLA, PCL, PCLA, Pluronic F-127, collagen gel, polypropylene, polytetrafluoroethylene	PCL, PLGA, cellulose-based hydrogels, alginate-based hydrogels, type 1 and 2 collagen hydrogel	PLA, PGA, collagen, gelatin, keratin, fibronectin, alginate, cellulose, HA, chitosan, GAG, human fibrin, PU, chitosan, PRP
Scaffold Manufacturing	Decellularized donor trachea, injection molding, electrospinning, 3D bioprinting	3D bioprinting	Injection molding, photopolymerization hydrogel system, external stenting, stereolithography
Current Human Applications	Repair of circumferential tracheal defects, repair of bronchial defects	Repair of LLC and ULC defects	Auricular cartilage framework for microtia repair
Current Limitations	Poor revascularization after implantation, lack of cellular ingrowth after implantation, inability to fully monitor graft after implantation, exposure to microbial organisms after implantation, recurrent granulation tissue formation and luminal collapse at short-term follow-up, anastomotic breakdown and fistulae formation at long-term follow-up	Lack of studies investigating human use, lack of FDA guidance on bioengineered nasal cartilage, large amount of heterogeneity in manufacturing methods limiting large-scale translation	Low chondrocyte cell numbers from initial donor cell harvest, lack of long-term follow-up studies, potential cytotoxic effects of scaffold breakdown products

Abbreviations: MSC—mesenchymal stem cell, PGA—polyglycolic acid, PLA—polylactic acid, PCL—polycaprolactone, PCLA—poly-l-lactide, PLGA—polylactic-co-glycolic acid, PU—polyurethane, HA—hyaluronic acid, GAG—glycosaminoglycan, PRP—platelet-rich plasma, LLC—lower lateral cartilage, ULC—upper lateral cartilage, FDA—Federal Drug Administration.

## Data Availability

No new data were created for this review.
